# Combinational Recommendation of Vaccinations, Mask-Wearing, and Home-Quarantine to Control Influenza in Megacities: An Agent-Based Modeling Study With Large-Scale Trajectory Data

**DOI:** 10.3389/fpubh.2022.883624

**Published:** 2022-05-26

**Authors:** Hao Zhang, Ling Yin, Liang Mao, Shujiang Mei, Tianmu Chen, Kang Liu, Shengzhong Feng

**Affiliations:** ^1^Shenzhen Institute of Advanced Technology, Chinese Academy of Sciences, Shenzhen, China; ^2^University of Chinese Academy of Sciences, Beijing, China; ^3^Department of Geography, University of Florida, Gainesville, FL, United States; ^4^Shenzhen Center for Disease Control and Prevention, Shenzhen, China; ^5^State Key Laboratory of Molecular Vaccinology and Molecular Diagnostics, School of Public Health, Xiamen University, Xiamen, China; ^6^National Supercomputing Center in Shenzhen, Shenzhen, China

**Keywords:** influenza, agent-based model, intervention measures, vaccinations, mask-wearing, home-quarantine, post-COVID-19 era

## Abstract

The outbreak of COVID-19 stimulated a new round of discussion on how to deal with respiratory infectious diseases. Influenza viruses have led to several pandemics worldwide. The spatiotemporal characteristics of influenza transmission in modern cities, especially megacities, are not well-known, which increases the difficulty of influenza prevention and control for populous urban areas. For a long time, influenza prevention and control measures have focused on vaccination of the elderly and children, and school closure. Since the outbreak of COVID-19, the public's awareness of measures such as vaccinations, mask-wearing, and home-quarantine has generally increased in some regions of the world. To control the influenza epidemic and reduce the proportion of infected people with high mortality, the combination of these three measures needs quantitative evaluation based on the spatiotemporal transmission characteristics of influenza in megacities. Given that the agent-based model with both demographic attributes and fine-grained mobility is a key planning tool in deploying intervention strategies, this study proposes a spatially explicit agent-based influenza model for assessing and recommending the combinations of influenza control measures. This study considers Shenzhen city, China as the research area. First, a spatially explicit agent-based influenza transmission model was developed by integrating large-scale individual trajectory data and human response behavior. Then, the model was evaluated across multiple intra-urban spatial scales based on confirmed influenza cases. Finally, the model was used to evaluate the combined effects of the three interventions (V: vaccinations, M: mask-wearing, and Q: home-quarantining) under different compliance rates, and their optimal combinations for given control objectives were recommended. This study reveals that adults were a high-risk population with a low reporting rate, and children formed the lowest infected proportion and had the highest reporting rate in Shenzhen. In addition, this study systematically recommended different combinations of vaccinations, mask-wearing, and home-quarantine with different compliance rates for different control objectives to deal with the influenza epidemic. For example, the “V45%-M60%-Q20%” strategy can maintain the infection percentage below 5%, while the “V20%-M60%-Q20%” strategy can maintain the infection percentage below 15%. The model and policy recommendations from this study provide a tool and intervention reference for influenza epidemic management in the post-COVID-19 era.

## Introduction

The response to severe acute respiratory syndrome coronavirus 2 (SARS-CoV-2) triggered new thinking on how to deal with respiratory infectious diseases (1), such as seasonal influenza, which has led to widespread morbidity and mortality worldwide (2–4). Intense interactions between individuals in megacities, such as those between adults working long hours in confined office buildings, often fostered and amplified the spread of influenza (5), threatening the health of people, particularly the elderly and children. Owing to the superimposed infection of influenza and other ongoing epidemics such as COVID-19 (6), it has become more important to control the influenza epidemics in megacities and maintain its spread at a low level. The course of influenza is self-limiting, and patients without complications often can recover in a few days. As a result, many cases have been undocumented (7–9). Due to low reporting rates on influenza and less detailed epidemiological investigations (9), it has been difficult to completely understand some critical and spatiotemporal characteristics under a micro scope of influenza transmission in megacities, such as total number of infections, the age distribution of the whole influenza-infected population, the reporting rates of different age groups, when and where individuals are infected, and whether there are other strong influenza transmission places in addition to homes and schools. These questions are of great significance in understanding the spread of influenza and formulating influenza intervention strategies for city government. Moreover, the human movements and interactions play critical roles in the transmission of influenza in urban environment (5). However, few studies investigated the influenza transmission characteristics based on human mobility of megacities in both the population and space-time dimensions at a fine scale (10). First, population-based compartment models cannot reflect the heterogeneity of individual attributes and are not flexible to model spatiotemporal characteristics at an fine scale within cities. Second, although some individual-level transmission models such as agent-based models integrate trajectory data to simulate the human mobility, the model's accuracy is rarely verified (11–13). Even if several individual-level transmission models for urban areas constructed by fusing trajectory data verified the model accuracy, either the specific accuracy indicators were not given (14), or they verified it at a coarse spatial scale (15, 16). Therefore, to answer these questions and uncover the black box of the influenza epidemic in a megacity, we need a finer-scale influenza transmission model such as a spatially explicit agent-based model that can reflect the heterogeneity of individual attributes, different types and places of contact activity, and the reality of intra-urban mobility.

Before the outbreak of COVID-19, vaccinations of the elderly and children (17–19), and school interventions (20–22) were the key measures to mitigate the spread of seasonal influenza. When the supply of influenza vaccines was limited, vaccine distribution strategies were generally designed based on a series of social and political factors and individuals with high mortality rates were usually prioritized. Although considering the mortality of different age groups is important, other important factors affecting the influenza transmission such as the age distribution of the urban population and the contact intensities between different ages should also be considered under influenza vaccine distribution (23). Moreover, since the onset of COVID-19, public awareness on vaccination, mask-wearing, and self-health management (such as self-isolation at home after contracting a fever) has generally increased for many countries and regions. To control the influenza epidemic and reduce the proportion of infected people with high mortality without considering intensive intervention measures (such as school closure), the combination of the three measures listed above should be carefully considered and quantitatively evaluated based on the fine-scale spatiotemporal transmission characteristics of influenza in megacities.

Therefore, with the aid of large-scale trajectory data and multi-source spatiotemporal urban data, this study proposes a spatially explicit agent-based model to simulate the influenza transmission between millions of individuals in a typical megacity, Shenzhen in China. Then, this model is used to systematically recommend the combination of vaccinations, mask-wearing and home quarantine toward to different influenza control objectives. Considering Shenzhen as a research area, the proposed model integrating large-scale anonymous mobile phone location data could finely describe individuals' travels and the interactions between individuals under different activity types including staying at home, working at workplace, studying at school and other activities. In addition, the model simulation accuracy was evaluated on a multiple intra-urban spatial scale based on confirmed influenza case data. Systematic recommendations for influenza intervention strategies have been made based on the age structure and spatial transmission characteristics of influenza transmission in cities. The results of this study provide a reference for influenza epidemic prevention and control in post-COVID-19 societies.

## Materials and Methods

### Study Area and Data

**Figure 1:**
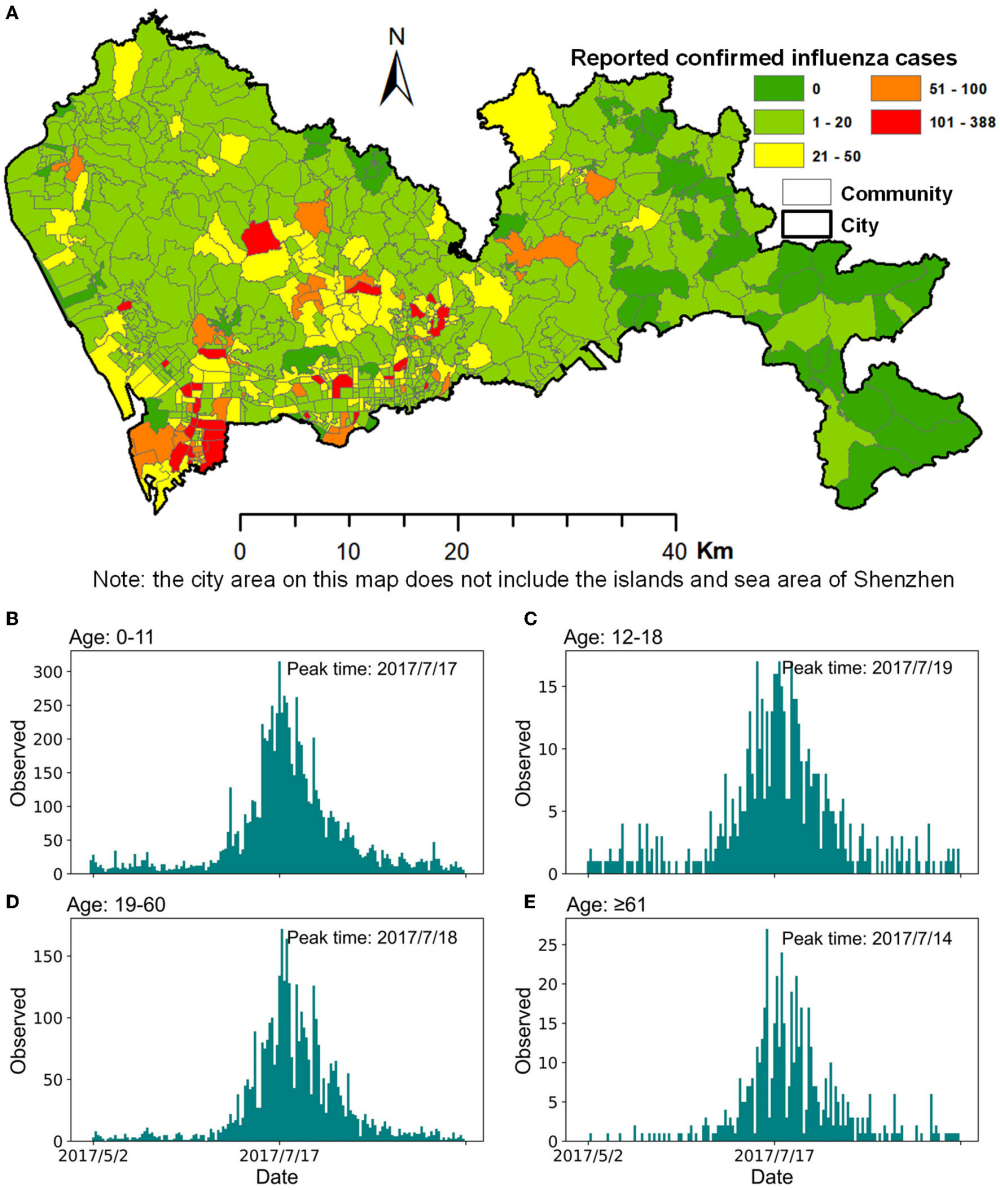
Confirmed cases of influenza in Shenzhen during the summer season of 2017. **(A)** Spatial distribution of confirmed influenza cases at a community scale. **(B–E)** Daily reported confirmed influenza cases in different age groups.

This study considers Shenzhen, a megacity in China, as the research area and explores a new plan for influenza intervention after COVID-19 based on a spatially explicit agent-based influenza model. Shenzhen (10 districts, 74 sub-districts, and 673 communities) is a major coastal megacity with more than 10 million people in southern China, adjacent to Hong Kong, in the Guangdong Province, covering an area of 1,997.47 km^2^ (Figure 1A).

In this study, to build the whole population agent model of Shenzhen, we first synthesized all the individuals according to the urban population size and age structure based on census data, and then assigned each individual to the corresponding family based on the family information in the travel survey data. Then, the individuals' home and work addresses were obtained by mining mobile phone location data and travel survey data, and the individuals' travel chains were reconstructed according to these two pieces of trajectory data. Finally, details such as the individuals' family addresses, workplaces, and locations in the activity chains were matched with the corresponding buildings according to their functions and locations in the building census data. The description of the above data can be found in the Supplementary Material.

#### Individual Trajectory Data

Mobile phone location data include 16 million anonymized mobile phone users of China Unicom on a typical working day in Shenzhen in 2012, which is composed of three parts, namely, the unique identifier for an anonymous mobile phone user, the longitude and latitude of the connected cell tower, and the timestamp when the location is recorded. After data cleaning, 5.8 million users with complete 24-h records during the day were selected. The service radius of the Shenzhen cell tower is between 200 m and 2 km. The major movement flows of mobile phone users in Shenzhen are shown in Supplementary Figure 1.

The travel survey data of Shenzhen in 2010 included 190,000 people, 220 thousand person-times of trips, and 11 types of activities. The spatial scale of the location was the traffic analysis zone. The dataset includes home address, household structure, income, work address, and travel information (e.g., travel purpose, trip origin and destination, and travel mode) for 98 thousand households.

#### Influenza Data

Shenzhen usually experiences two seasonal influenza outbreaks in summer and winter, and has enhanced its comprehensive influenza surveillance since 2017. In our study, we selected data on the summer influenza from Shenzhen in 2017 as the research data because it has a complete epidemic outbreak cycle. The influenza data with a total of 14,473 confirmed cases (from May 1 to September 30) collected by the Shenzhen Center for Disease Control and Prevention contained information on the outpatient cases of hospitals and community health service centers (Figure 1). Influenza A (H3) was the dominant strain in this seasonal influenza epidemic. This case data included each patient's unique identification number, age, onset time, diagnosis time, name of the hospital or community health service center, and the patient's home location. The overall peak of this influenza season was July 17. After grouping the reported confirmed cases by age, namely 0–11, 12–18, 19–60, and ≥61, the peak times of influenza for the four age groups were July 17, 19, 18, and 14 (Figure 1), respectively.

### Research Framework

**Figure 2:**
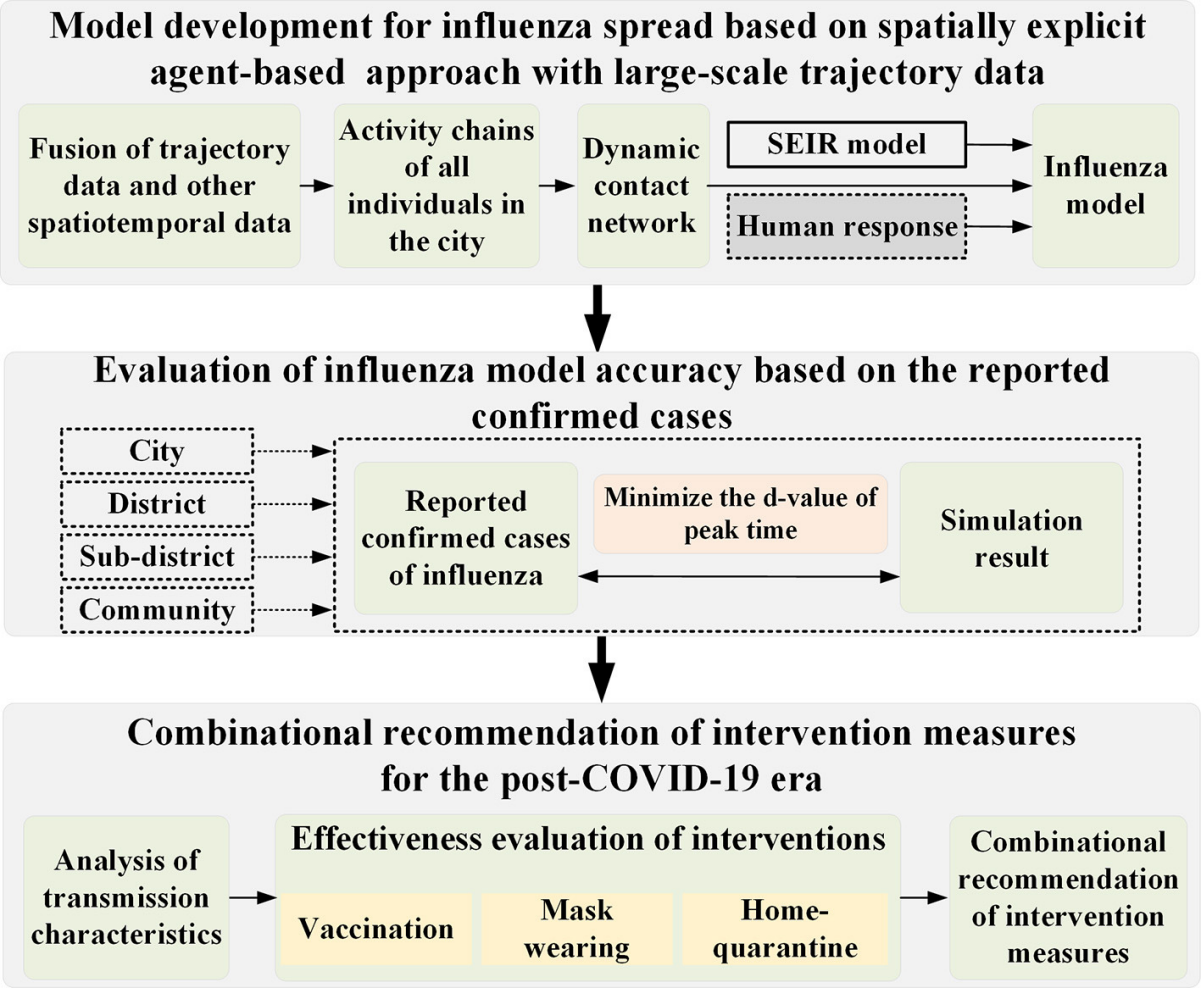
The overall research framework of this study.

The framework of this study is illustrated in Figure 2. To develop a spatially explicit agent-based influenza model, multi-source urban data were integrated to build dynamic contact networks between individuals in the city, and home-quarantining by individuals at different ages was considered to reflect their response actions to the onset of influenza symptoms. The model simulation accuracy was evaluated based on the spatiotemporal distribution of the observed case data at four spatial scales including city, district, sub-district and community. After understanding the transmission characteristics of influenza for different ages and activity types, interventions to slow the spread of influenza and reduce the infection proportion of people with high mortality were recommended. These three components of the study are introduced in detail in the following sections.

The proposed agent-based model considers a single individual as an agent to be the smallest modeling unit, and the intervention measures are also simulated at the individual level; finally, the model recommends intervention measures for targeted people of high risks. In terms of time dimension, the agent-based model we developed simulates individual activities for 24 h a day for the 5-month influenza transmission process in the city. For the spatial dimension, the activities of individuals in the model were simulated with explicit building locations, which allows identifying influenza transmission locations and making risk maps at flexible intra-urban scales.

### Spatially Explicit Agent-Based Influenza Model

**Figure 3:**
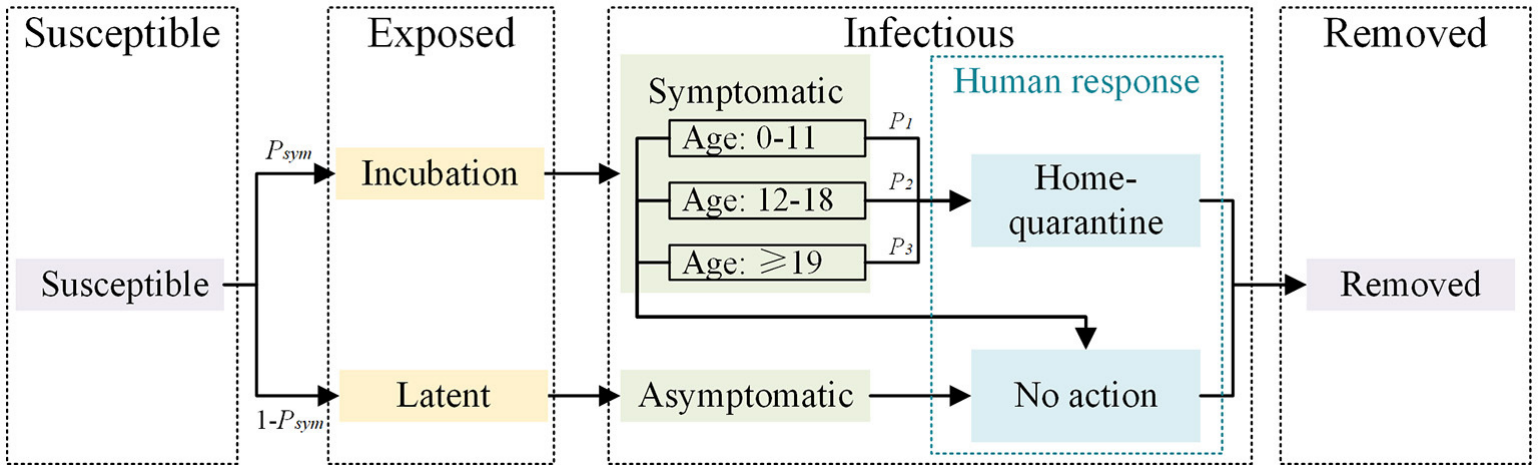
The SEIR model integrating human response.

The proposed framework of the spatially explicit agent-based influenza model consists of three parts. First, large-scale individual trajectory data and statistical data were fused to develop the individual mobility model; details on this can be found in a previous study (15). The synthetic individuals had both demographic attributes (e.g., age, gender, family structure, and workplace) and activity chains (further details in the Supplementary Material). The activity chain used the hour and building as a unit to record the locations where individual activities were performed. These activities were divided into four types (e.g., home, work, school, and others). Second, a dynamic contact network based on an individual activity chain was built; details can be also found in a previous study (15). Individuals performing the same activities at the same hour in the same buildings were marked as individuals with spatiotemporal co-occurrences. The individuals were divided into multiple fixed contact groups based on their spatiotemporal co-occurrences at home, work, and school. Interactions between individuals in the same fixed contact groups were regular encounters, and those in different fixed contact groups were random encounters; fixed contacts represent acquaintances and random contacts represent strangers. Third, considering that individuals infected with influenza would have different degrees of symptoms and display certain active response behaviors (such as dropping out of school), the response action of home-quarantining was integrated into the SEIR (susceptible-exposed-infectious-removed) model (Figure 3) and the spread of influenza on the contact network was simulated.

To quantify the heterogeneity of individuals' responses to influenza, the incubation, latent and infectious periods of infected individuals and symptomatic individuals withdrawing to home were set as probability events. The lengths of incubation, latent, and infectious periods in the model are shown in Table 1 (24–26). The proportions of home-quarantine individuals of different age groups were expressed as *P*_1_, *P*_2_, and *P*_3_. The parameters of home-quarantine after the onset of influenza are shown in Table 2 (25). Based on the types of activities and human contact patterns, the intensities of daily contact *I*_*c*_ at different contact settings (15) were assigned to individuals with spatiotemporal co-occurrences (further details in the Supplementary Material). After the incubation period, infectious individuals developed symptoms with a probability *P*_*sym*_ = 67% (25–27), and the relative infectivity *r* of the symptomatic and asymptomatic cases was 1.0 and 0.5 (25, 26, 28), respectively. Finally, the probability of an individual being infected was *P* = *pTrans* × *I*_*c*_ × *r*, where *pTrans* was the transmission probability per contact. In addition, the model set the proportion of people with immunity to 30%. The sensitivity analysis of the immune proportion is shown in the Supplementary Material. To reduce the influence of model uncertainties on the simulation results, all the experiments in this study were simulated 100 times. Because of the long-tail distribution of simulation results, the median value of the results was used for further analysis.

**Table 1:** The duration and probability distribution of exposed and infection period in the model.

**Period**	**Duration and probability**					
	**1 d**	**2 d**	**3 d**	**4 d**	**5 d**	**6 d**
Incubation/Latent	30%	50%	20%	0	0	0
Infectious	0	0	30%	40%	20%	10%

**Table 2:** The parameters of home-quarantining after onset of influenza.

**Age groups**	**Home–Quarantine proportion**	**Delay time and probability of home–quarantining after onset**		
		**0 d**	**1d**	**2 d**
0–11	*P_1_*	0.27	0.53	0.20
12–18	*P_2_*	0.30	0.58	0.12
19–100	*P_3_*	0.20	0.60	0.20

### Model Performance Evaluation

Before exploring the spatiotemporal transmission characteristics of influenza in a city, we evaluated the simulation accuracy of the proposed model at multiple intra-urban spatial scales. Owing to the large number of undocumented influenza cases, there was no ground truth of the entire infections. However, the observed peak time of the epidemic curve usually represented the real peak time (5, 29), and accurately simulating the epidemic curve knee point is of great significance to the development of infectious disease prevention and control. Therefore, the model accuracy was evaluated by the time difference between the simulated and observed peak time in each spatial unit at different spatial scales based on the standardized daily cases and simulated cases, as shown in Equation (1):


(1) $$\{\begin{array}{c} \begin{array}{c} \Delta_{peak,i}=|\;T_{s,i}-T_{r,i}\;|,\;i=1,\;2,\ldots ,n,\\ \Delta_{peak,m}=median\left(\Delta_{peak,i}\right),\;i=1,\;2,\ldots ,n,\\ Sum_{\Delta }=\Delta_{city}+\;\Delta_{district}+\;\Delta_{sub-district}+\Delta_{community}, \end{array}\\ Std_{\Delta }=std\left(\Delta_{city},\Delta_{district},\Delta_{sub-district},\Delta_{community}\right), \end{array}$$

where, n is the number of spatial units under a specified spatial scale; *T*_*s, i*_ *and T*_*r, i*_ are the peak times of the simulated and reported epidemic curves in the i^th^ spatial unit, respectively; Δ_*peak, i*_ is the absolute value of the d-value between the simulated and reported peak time in the i^th^ spatial unit; Δ_*peak, m*_ is the median value of Δ_*peak, i*_ under the specified spatial scale; Δ_*city*_, Δ_*district*_, Δ_*sub*−*district*_, Δ_*community*_ are the Δ_*peak, m*_ values under the four spatial scales of city, district, sub-district, and community, respectively; and *Sum*_Δ_ and *Std*_Δ_ are the sum and standard deviation of Δ_*city*_, Δ_*district*_, Δ_*sub*−*district*_, and Δ_*community*_.

Before evaluating the simulation accuracy of the model, four unknown parameters in the model were calibrated: *pTrans*, the transmission probability per contact; *P*_1_, *P*_2_, and *P*_3_, the proportions of individuals 0–11, 12–18, and ≥19 years old that quarantined at home after onset, respectively. The model parameter calibration criteria developed in this study are as follows. (1) At the city scale, the effective reproduction number *R*_*eff*_ ranges from 1.1 to 2.0 (28, 30–32). (2) At each spatial scale (city, district, sub-district, and community), the Δ_*peak, m*_ should be as small as possible, while the highest priority is given to the city scale, followed by the other three scales. (3) Usually, children are likely to be taken care of at home when they do not feel well, while adults tend to not stay at home unless they are really sick. This study attempted to prove that the older the age group, the lower the proportion of individuals who home quarantined (i.e., *P*_1_≥*P*_2_≥*P*_3_), and a set of parameters with the minimum Δ_*city*_ value was preferentially selected.

Following the calibration of the model parameters, the simulation accuracy of the model was evaluated at multiple spatial scales using the two steps listed below. First, the number of reported cases, population size, and the Δ_*peak, i*_ values in each spatial unit at each spatial scale were calculated. Second, the proportion of spatial units, *P*_*s*_, the proportion of the population, *P*_*p*_, and the proportion of reported cases, *P*_*r*_, in those spatial units whose Δ_*peak, i*_ ≤ *T* (*T* = 1, 2,…,6 d) under each scale were calculated. This study used *T* = 6 as the criterion because the maximum length of the infection period was 6 d. The related calculation method is shown in Equation (2):


(2) $$\{\begin{array}{c} P_{s}=\frac{N_{s}}{total\;number\;of\;spatial\;units},\\ P_{p}=\frac{N_{p}}{total\;population},\\ P_{r}=\frac{N_{r}}{total\;number\;of\;cases}, \end{array}$$

where *N*_*s*_ is the number of spatial units with Δ_*peak, i*_ ≤ T days, *N*_*p*_ is the population size of these spatial units, and *N*_*r*_ is the number of reported cases in these spatial units. The more spatial units, population sizes, and cases covered, the higher the simulation accuracy of the model at that scale.

### Analysis of Influenza Transmission Characteristics in the Study Area

The development of targeted interventions was based on the characteristics of influenza transmission within the study area. The epidemiological characteristics of influenza were sensitive to age, rather than sex, in terms of demographic characteristics. We also needed to update our understanding on locations where the exposure events happen (such as homes, workplaces, and schools) due to the changing lifestyle in megacities caused by the well developed urban transportation system, long working hours, and the diverse daily activities. However, because of the large number of undocumented cases and the difficulty in investigating the exposure sites of reported cases, important attributes such as the age and exposure locations of the actual infected individuals were not clear. Given that the proposed model could represent patients' ages and exposure locations under the spread of influenza, this study analyzed the influenza transmission characteristics from these two important aspects.

### Intervention Effectiveness Evaluation and Combination Recommendation

This study focused on evaluating the effectiveness of different combinations of vaccinations, mask-wearing after onset for out-of-home activities with other people, and home quarantining after onset, which are suggested as effective means to control acute respiratory epidemics during the COVID-19. Vaccinations were generally completed before the influenza season. Individuals who had been vaccinated and had sufficient antibodies would not likely be infected during the epidemic. To maintain the R_eff_ below 1, the coverage of vaccination was calculated based on the relationship between herd immunity and the effective reproduction number of influenza (i.e., 1.1~2.0) (33). As a result, in our simulation, the coverage of vaccination was set as 10–50% of the target population, gradually increasing in steps of 5%, for a total of nine groups of parameters, and the influenza vaccine effectiveness for susceptibility was set as 50% according to previous studies (34–36). The effectiveness of wearing masks in inhibiting the spread of influenza virus was set at 70% (37–39). The mask-wearing proportion was 0–100%, which increased gradually in steps of 20% for a total of six groups of parameters in our simulation. The parameter settings of home-quarantine after onset are shown in Table 2 for the baseline scenario; individuals that quarantined at home only interacted with family members. In the scenarios of intervention recommendation, we only simulated a higher home-quarantine proportions than those of the baseline scenario.

## Results

### Calibration of Unknown Model Parameters

Based on the distribution of influenza case data at different spatial scales, we first calibrated four unknown parameters (*pTrans, P*_1_, *P*_2_, and *P*_3_) in the model (Table 3). The value of *pTrans* was limited by the effective reproduction number from 1.1 to 2.0. When selecting the values of *P*_1_, *P*_2_, and *P*_3_, this study ranked the parameter groups based on the Δ_*peak, m*_ value from city to community scale. “1.0_0.6_0.2” was finally determined as the proportion of infected individuals from each age group that quarantined at home, because the *Sum*_Δ_ and *Std*_Δ_ values were the smallest and the relationship among *P*_1_, *P*_2_, and *P*_3_ satisfied *P*_1_ ≥ *P*_2_ ≥ *P*_3_, which is called the baseline scenario. Here, *pTrans* = 2.5, *R*_*eff*_ = 1.59, and the infection percentage in population was 35.62%.

**Table 3:** Calibration of the unknown model parameters.

**Parameters (*P_**1**_*_*P_**2**_*_*P_**3**_*)**	**Δ_*peak, m*_** **(d)**						***R*_*eff*_**
	**Δ_*city*_**	**Δ_*district*_**	**Δ_*sub–district*_**	**Δ_*community*_**	***Sum*_Δ_**	***Std*_Δ_**	
0.0_0.2_0.6	0	4	11	14	29	5.54	1.30
0.6_0.2_0.4	0	5	7	12	**24**	4.30	1.70
0.0_0.6_0.4	0	6	9	13	28	4.74	1.79
0.8_0.0_0.4	0	7	7	12	26	4.27	1.88
0.6_0.8_0.2	1	4	7	12	**24**	4.06	1.71
**1.0_0.6_0.2**	1	4	7	12	**24**	4.06	1.59
0.4_0.4_0.4	1	4	8	13	26	4.50	1.62
0.8_0.8_0.2	1	5	7	12	25	3.96	1.49
0.8_0.2_0.4	1	5	9	12	27	4.15	1.94
0.4_1.0_0.2	1	6	7	12	26	3.91	1.82
0.8_0.6_0.2	1	6	7	12	26	3.91	1.65

*Note: Only part of all parameter groups (6 × 6 × 6 = 216 groups in total) are listed here to illustrate the parameter calibration process. The bold value of parameters (P1_P2_P3) is the selected one for model calibration, while the bold values of Sum_Δ_ are the minimum ones*.

### Evaluation of Influenza Model Simulation Accuracy

**Figure 4:**
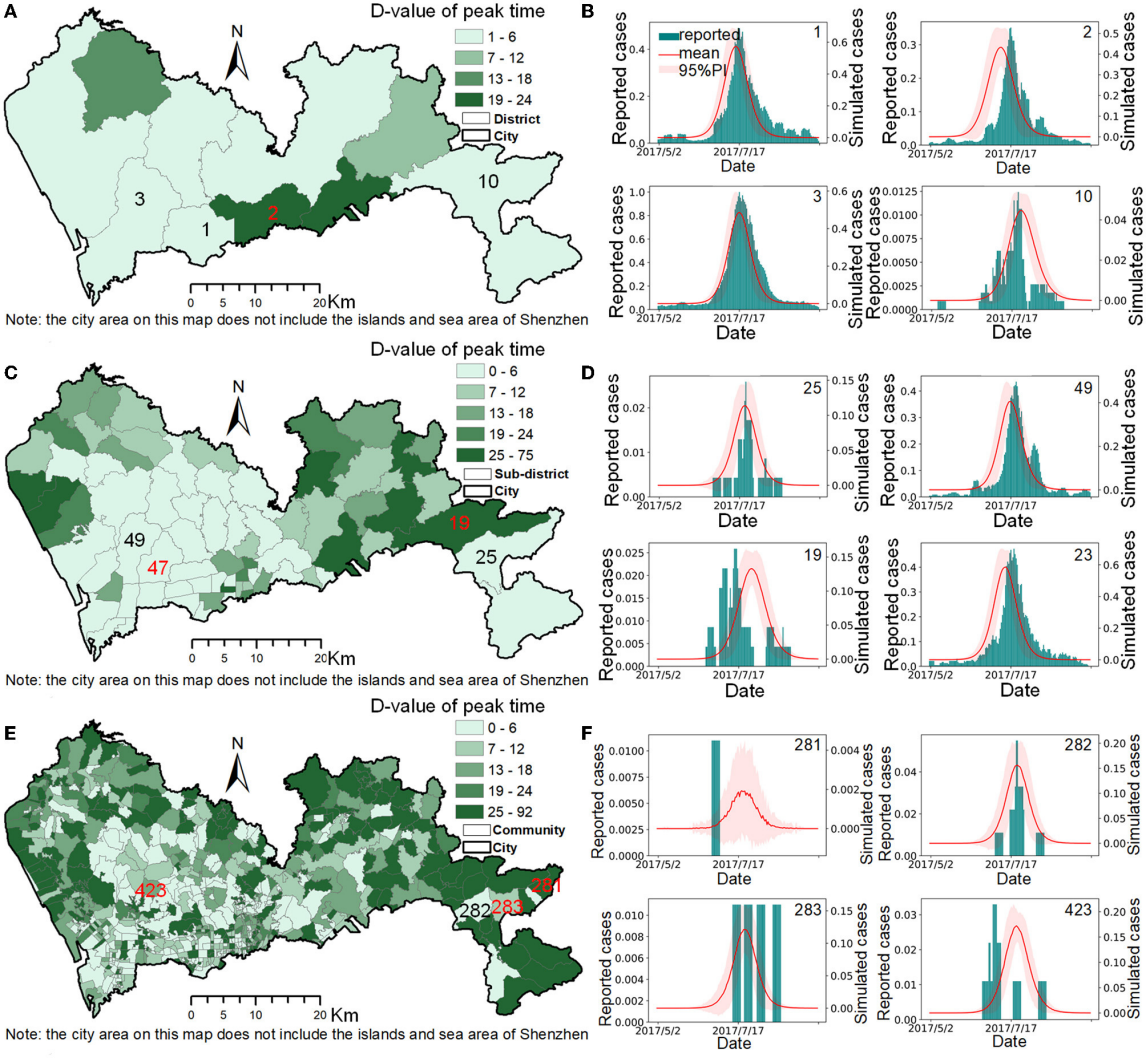
The model accuracy at different spatial scales. **(A,C,E)** are the spatial distributions of the Δ_*peak*_ values at the district, sub-district, and community scales. **(B,D,F)** are the comparisons between the simulation cases and reported cases of selected typical spatial units at the district, sub-district, and community scales.

In this study, the proportion of spatial units, *P*_*s*_, reported cases, *P*_*r*_, and population size, *P*_*p*_ covered by Δ_*peak, i*_ ≤ T (T=1, 2, 3…6 days) at the district, sub-district, and community scales were used to evaluate the model's simulation accuracy at different spatial scales (Table 4). Taking the maximum infection period of 6 d as the tolerance standard for Δ_*peak, i*_, the results showed that the simulation accuracy of the model decreased as the spatial scale becomes finer. At the district scale, the simulation accuracy of the model was relatively high, with the median of the Δ_*peak, i*_ being 4 d, and the Δ_*peak, i*_ ≤ 6 d covered 60% of the spatial units (Figure 4A), 79.3% of the population, and 86.0% of the reported cases. At the sub-district scale, the simulation accuracy of the model was acceptable. The median of the Δ_*peak, i*_ was 7 d and the Δ_*peak, i*_ ≤ 6 d covered 67.7% of the reported cases and 53.8% of the population in 47.3% of the spatial units (Figure 4B). The simulation accuracy of the model was slightly lower at the community scale, with the median of the Δ_*peak, i*_ being 12 d. Only 45.6% of the communities (Figure 4C) had a Δ_*peak, i*_ ≤ 6 d, and only ~32% of the case data and urban population were included. The small size of reported cases in some communities was the main reason for the low simulation accuracy at the community scale (Figure 4F). Taking Figure 4 as an example, for the two spatial units with identifiers of 281, 283, which belong to spatial unit 25 at the sub-district level, it was difficult to verify the simulation accuracy because of the sparseness of the reported case data.

**Table 4:** The accuracy of the proposed influenza model at three different intra-urban scales.

**Spatial scale**	**Accuracy** **indictor**	**Δ**_**peak, i**_ **(d)**					
		**≤1 %**	**≤2 %**	**≤3 %**	**≤4 %**	**≤5%**	**≤6 %**
District	*P*_*s*_	30	30	40	50	50	60
	*P*_*r*_	44.3	44.3	61.7	72.3	72.3	86.0
	*P*_*p*_	23.3	23.3	36.2	59.3	59.3	79.3
Sub-district	*P*_*s*_	9.5	13.5	24.3	35.1	43.2	47.3
	*P*_*r*_	15.5	22.7	41.3	57.4	65.8	67.7
	*P*_*p*_	11.4	16.6	26.6	42.4	49.6	53.8
Community	*P*_*s*_	12.5	19.5	26.0	36.2	39.0	45.6
	*P*_*r*_	9.8	14.3	18.4	24.8	28.2	32.4
	*P*_*p*_	8.0	12.9	18.0	24.3	28.0	32.1

*P_s_ is the proportion of the spatial units, P_r_ is the proportion of the reported cases, and P_p_ is the proportion of the population size*.

### Influenza Transmission Characteristics

**Figure 5:**
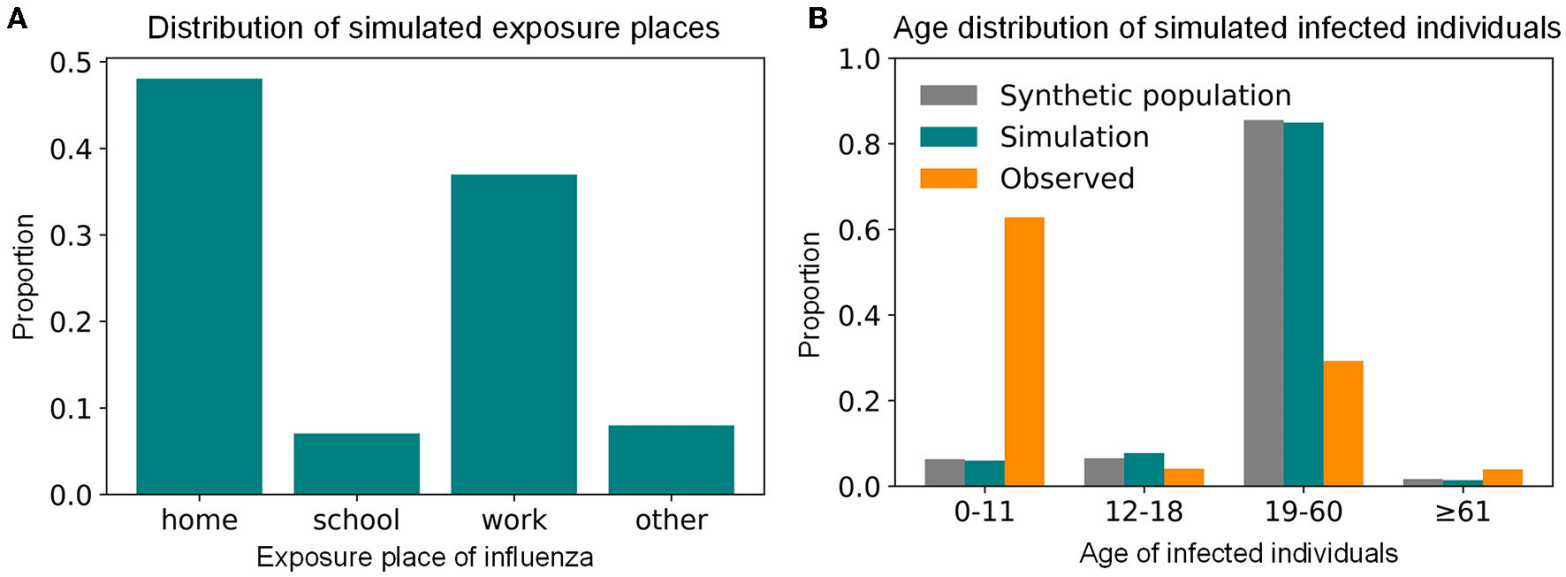
The simulated transmission places and age distribution of influenza-infected individuals during the summer seasonal influenza epidemic of 2017 in Shenzhen. **(A)** The distribution of simulated transmission places. **(B)** The age distribution of simulated infected individuals.

In the simulation results of the baseline scenario (i.e., the summer influenza season in 2017), the influenza transmission in Shenzhen was mainly within the family, followed by the workplace, other places, and finally the school (Figure 5A). This showed that the workplace, where urban individuals worked for a long time and had many contacts, was also an important place for the spread of influenza, which not only confirms common sense, but also emphasizes the importance of intervention for working people. The age distribution of the influenza-infected individuals in the simulation did not agree with the reported data (Figure 5B). Our results suggested that many children aged 0–11 would seek medical help after onset and had a relatively high reporting rate of 3.82%, while adults with influenza were not likely to seek medical help, leading to a lower reporting rate of 0.13%. In other words, due to physical endurance and parental care, many infected children will choose hospitals for treatment after onset, while the proportion of infected adults seeking medical care is relatively small, resulting in a high proportion of children in the influenza surveillance system. However, as our model suggested, there could be many undocumented infected adults who played an important role in the influenza transmission. This finding implied that in addition to paying attention to the elderly and children, who have been targets of concern for a long time, we should also consider adult intervention measures to more effectively control the impact of influenza on key populations.

### Recommended Combination of Intervention Measures

**Figure 6:**
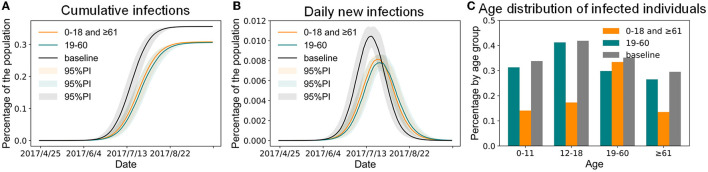
The impact of vaccinating the same population size in different age groups of Shenzhen. **(A)** Cumulative infections. **(B)** Daily new infections. **(C)** Age distribution of infected individuals.

According to simulations based on our model, in addition to paying attention to the elderly, children, families, and schools that have been groups of concern for a long time, attention should also be paid to the prevention and control of influenza in megacities among adults and in workplaces, which play an important role in the spread of influenza during the epidemic season. Therefore, this study first compared the impact of administering the same quantity of the vaccine to different age groups on controlling influenza outbreaks. Compared with the baseline scenario, both vaccination schemes reduced the infection size (Figure 6A) and delayed the peak time (Figure 6B). As Figure 6A shows, when the vaccine efficacy for susceptibility for influenza was 50% and ~1.6 million people aged 0–18 and ≥ 61 years old were vaccinated, the infection percentage of the urban population was 30.81%, and that of children and the elderly decreased significantly (Figure 6C). When the same amount of the vaccine was administered to adults aged 19–60 (i.e., 16.88% of them), 30.59% of the simulated individuals would eventually be infected with the infection percentage of the children and elderly decreasing slightly (Figure 6C). Compared with the baseline scenario, the two vaccine distribution schemes could only reduce the infection percentage by ~5%. In other words, when the number of vaccines was insufficient (there were only vaccines equivalent to the number of children and the elderly), regardless of how they were distributed, it was not enough to control the influenza epidemic. In addition, even if all the children and elderly were vaccinated, 14.1% of children and 13.5% of the elderly would still be infected (Figure 6C). Therefore, new intervention programs were required to further reduce the outbreak degree of influenza in Shenzhen.

Since our model simulations suggested that adults and workplaces played important roles in influenza transmission in Shenzhen, this study proposes an influenza intervention target strategy for adults based on the combination of the three interventions (vaccinations, mask-wearing, and home-quarantine). Effectiveness of three interventions is shown in the Supplementary Material. Considering that the mortality rate of children and the elderly in the influenza epidemic was significantly higher than that of adults, the influenza intervention program for adults proposed in this study was implemented after there was a complete vaccine coverage for children aged 0–11 and the elderly ≥ 61 years old.

**Figure 7:**
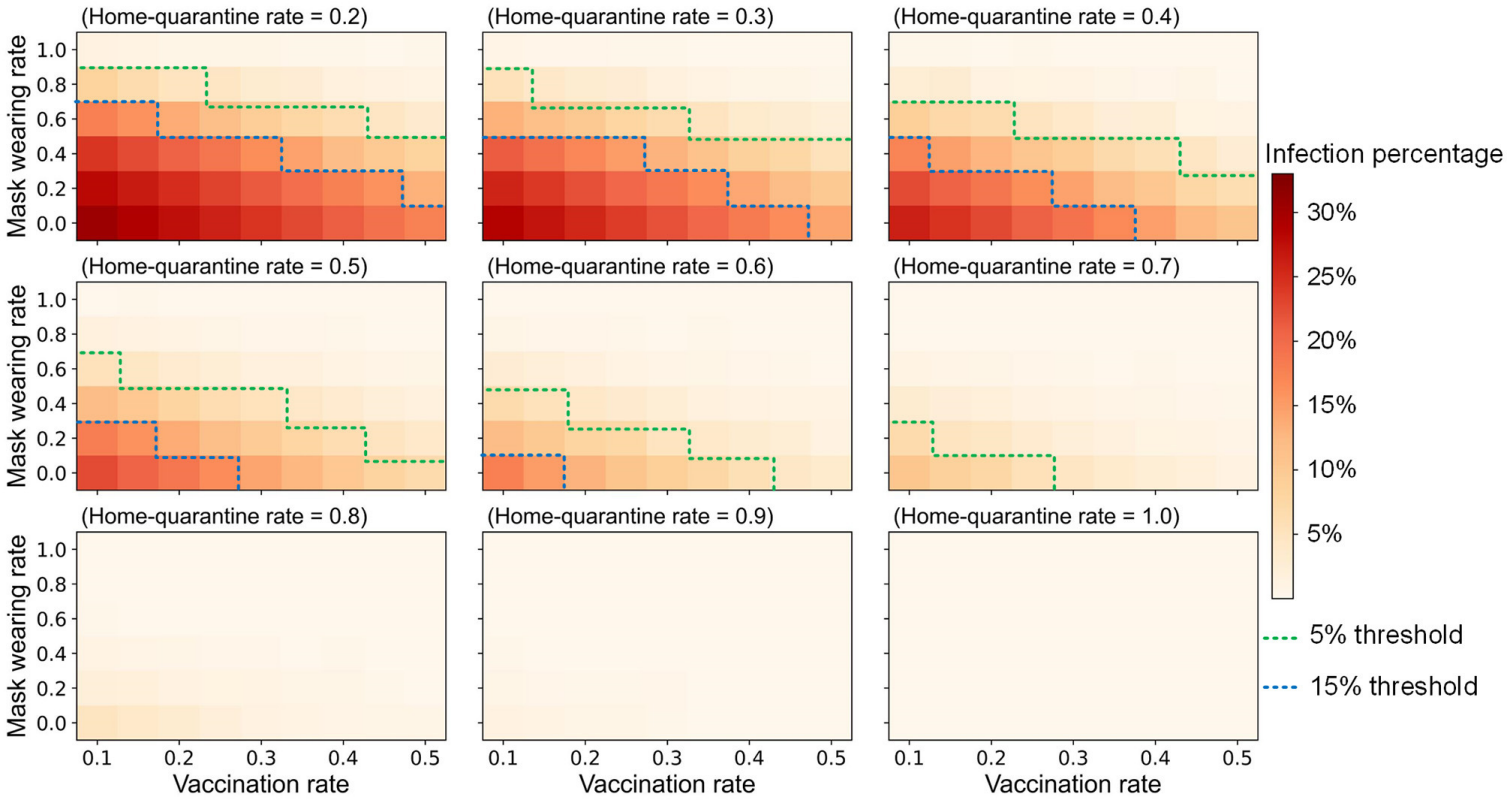
The influenza infection size of Shenzhen under systematical combinations of vaccination rate, mask wearing rate and home-quarantine rate for adults based on the premise that the children and elderly have been fully vaccinated. The green and blue dashed curves are the thresholds of 5% and 15% infection percentage, respectively.

**Figure 8:**
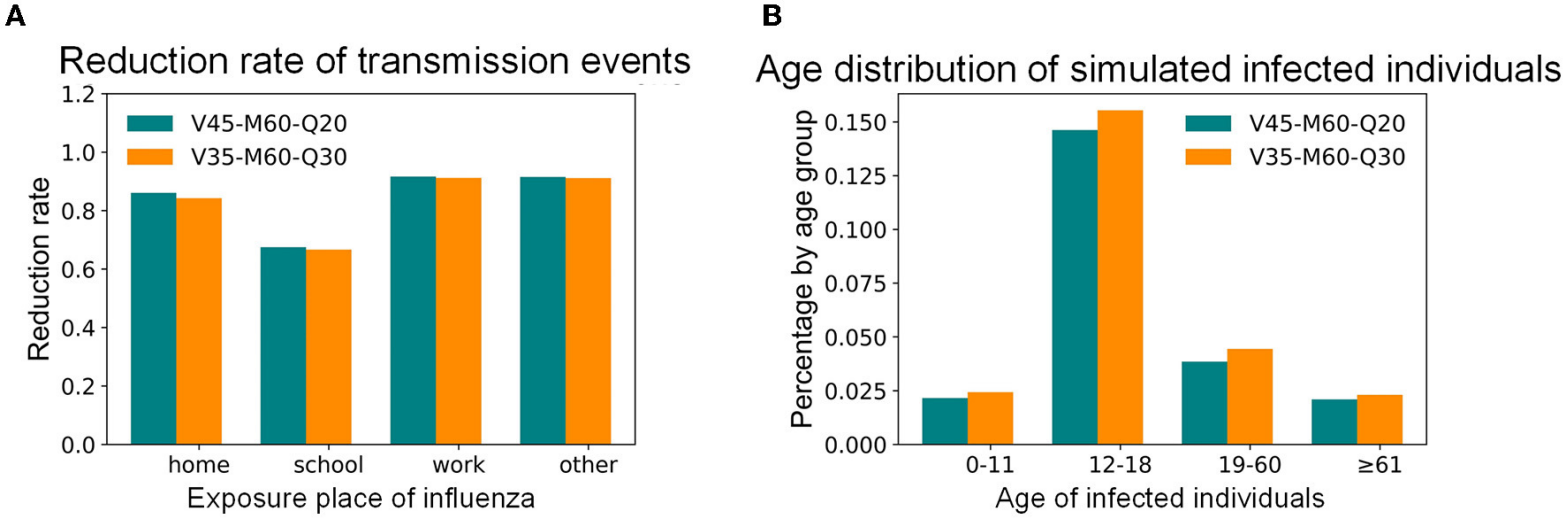
The simulated influenza transmission characteristics in Shenzhen under selected intervention combinations. **(A)** Distribution of simulated transmission places. **(B)** Age distribution of simulated infected individuals.

This study focused on the goals of controlling the infection percentage in Shenzhen and the infection proportion of people with high mortality for influenza epidemic prevention and control. The simulation results of vaccinations, mask-wearing, and home-quarantine for adults with different compliance rates are shown in Figure 7. For example, if the control objective is to maintain the infection percentage below 5%, which is a relatively high objective, 60% of infected adults after onset wearing masks for all activities except living at home during the influenza season, 20% of infected adults after onset staying home, and 45% of adults being vaccinated, can achieve this objective; this combination was recorded as the “V45%-M60%-Q20%” strategy. Under the same control objective, if the proportion of infected adults that quarantined at home after onset can also increase to 30%, the vaccination rate of adults can be decreased to 35%; this combination was denoted as the “V35%-M60%-Q30%” strategy. Compared with the 35.62% infection percentage in the baseline scenario, these two strategies reduced the infection percentage by 86%. However, when the rate of mask-wearing was low, it was necessary to increase the rates of vaccinations or home quarantine to effectively control the influenza epidemic. These intervention combinations effectively reduced the infection percentages in children, the elderly, homes, and workplaces while also reducing the whole urban infection percentage. The transmission events at home, school, and workplace have been reduced by 84, 67, and 91% (Figure 8A), and the infection percentages of children and the elderly have been both reduced from around 30% to around 2% (Figure 8B). Except for the above example with a 5% infection percentage as the control objective, Figure 7 also shows the combinations of the intervention measures corresponding to other control objectives for different control scenarios. For instance, if the control objective is to maintain the infection percentage below 15%, 60% of infected adults after onset wearing masks for all activities except living at home during the influenza season, 20% of infected adults after onset staying home, and 20% of adults being vaccinated, can achieve this objective; this combination was recorded as the “V20%-M60%-Q20%” strategy.

## Discussion

The COVID-19 pandemic, which has lasted more than 2 years, has changed the lifestyles of people worldwide. Measures such as mask-wearing, vaccination, nucleic acid detection, and home-quarantine implemented during the epidemic have become common interventions in most countries. These measures are do not extremely affect normal life and provide new intervention combinations for influenza prevention and control. Before the outbreak of COVID-19, most models suggested influenza interventions for children (17), the elderly (19), homes (40, 41), and schools (42–44), considering the decrease in susceptibility with age and the high mortality rate of children and the elderly (45). Our model found that vaccinations alone for children and the elderly were insufficient for reducing the urban infection percentage and the proportion of infected children and the elderly due to the contact between different age groups. This study systematically recommended combinations of vaccinations, mask-wearing, and home-quarantine under different compliance rates targeting for adults to deal with the influenza epidemic after the influenza vaccine was fully distributed for children and the elderly. Policymakers can choose different intervention schemes according to the diversity of abilities for urban epidemic prevention and control. The “V45-M60-Q20” strategy recommended by our model can reduce the urban infection size below 5% and that of children and the elderly to 2%. The model also encourages adults to withdraw to their homes after the onset of influenza to control its spread among individuals from the source. When the vaccine supply is insufficient, the mask-wearing rate or home-quarantine rate need to be increased. When the vaccination rate and mask-wearing rate are low, more infected adults need to be encouraged to work from home. Overall, increasing the supply of influenza vaccines and the vaccination rate, encouraging more adults to wear masks, and encouraging working from home with influenza infection risk are effective strategies to control the influenza epidemic in the post-COVID-19 era.

The COVID-19 pandemic has underscored the importance of accurate estimates of the size and duration of epidemics, which puts forward higher requirements for the accuracy of the epidemic model. This study systematically evaluated the simulation accuracy of the proposed spatially explicit agent-based influenza model at multiple intra-urban spatial scales. Previous studies mostly evaluated the simulation accuracy of the model at the city scale and considered the d-value between the peak times of the simulation results and case data as the evaluation standard (5, 29). The evaluation results of this study showed that the accuracy of the proposed agent-based spatially explicit model was good at the district and sub-district scales. One of the reasons for the low model accuracy at the community scale was that many spatial units did not have sufficient case data to evaluate the simulation accuracy.

Large-scale experiments based on our model have found that the infection percentage during the summer influenza season of Shenzhen in 2017 was 35.62%, indicating that there were many undocumented cases. The simulation results showed that adults were the most infected people in Shenzhen, but the reporting rate of adults was very low at ~0.13%, which is consistent with the range of reporting rates of 2009 H1N1 influenza in eight southern hemisphere countries (9). The infection percentage of children and the elderly, who have been groups of concern in the real-world, was lower than adults, while their reporting rates were relatively high at 3.8 and 1.03%, respectively. The simulated age structure of infected individuals was consistent with the entire population of Shenzhen (Figure 5B) and was also slightly related to the number of contact in each age group (Supplementary Figure 2C), indicating that the age structure and intensity of interaction were very important factors in the formulation of an intervention plan (17, 23). According to the analysis of the activity places where influenza was transmitted, the main transmission was within the family, which was consistent with previous studies (26, 41). Moreover, it showed that the workplace, where urban individuals worked for a long time and had a large number of contacts, was another major contributor to the spread of influenza in Shenzhen, and needed the attention of public health policy. These transmission characteristics that cannot be given by the traditional influenza surveillance system can be simulated by our high-precision, spatially explicit, agent-based influenza model.

This study had several limitations. First, this study selected only one megacity, Shenzhen, with a population of about 12 million (the age structure is dominated by adults) and emerging technology industries mostly located in confined office buildings, as the research area. The main influenza-infected population in the city was adults, and the main transmitters of influenza were families and workplaces. When extending the research results to other megacities, it is necessary to compare the population size, age structure, individual contact patterns at different ages, and industrial characteristics. Second, China's latest decennial census data of 2020 have not been released yet. Therefore, we used the 2010 census data. Similarly, a large-scale travel survey in Shenzhen occurs once every 10 years; this study used the travel survey data from 2010. To match the above data, this study used mobile phone location data and building census data from 2012. Although the above dataset was not the latest data for the study year of 2017, it still reflected the actual population distribution and travel patterns, so theoretically, it does not have a substantive impact on the conclusions of this study. Third, this study did not simulate the activities of individuals on weekends and holidays, which may have impacts on the contact networks. However, the recommended combination of different intervention measures can also be competent for the prevention and control of influenza. Fourth, this study did not exclude the spatial units with sporadic cases in the process of model accuracy evaluation to prove a better model accuracy because there is no clear criteria to discard the spatial units with small size of influenza cases. In the future, retrospective forecasts or out-of-sample predictions will be used to further validate the model.

## Conclusions

The contributions of this study can be summarized from the following three perspectives. First, a high-spatial-resolution influenza transmission model was built by integrating multi-source urban big data, and the model simulation accuracy was evaluated on the scales of the city, district, sub-district, and community with confirmed influenza data, offering a data-driven model with a relatively high accuracy at intra-urban scales. Second, based on our simulation results of the spatiotemporal characteristics of influenza transmission, this study proposed paying attention to the interventions for adults besides of children and the elderly. Third, according to the enhanced public awareness on vaccinations, mask wearing, and self-health management during the COVID-19 pandemic, the effects of different combinations of vaccinations, mask wearing, and home-quarantine rates on the influenza epidemic were systematically simulated, and the combination of these measures for different control objectives was recommended. The recommended intervention combinations for Shenzhen can also be scientific reference for many other megacities worldwide in the post-COVID-19 era.

## Data Availability Statement

The data analyzed in this study is subject to the licenses/restrictions as follows. These datasets cannot be shared directly because it needs the permission of relevant departments and it compromises patient privacy. However, researchers who meet the criteria for accessing confidential data can send requests to the local government departments. Mobile phone data were provided by the Shenzhen Transportation Operation Command Center (Contact: Binliang Li, 240854198@qq.com). Travel survey data, building survey data and census data were offered by the Planning and Natural Resources Bureau of Shenzhen Municipality (Contact: Renrong Jiang, jiangrenrong@126.com). The influenza data were provided by the Shenzhen Center for Disease Control and Prevention (Contact: SM, sjmei66@163.com).

## Author Contributions

LY, LM, SM, and HZ conceived and designed the study. HZ, LY, LM, TC, SM, KL, and SF developed the model. HZ programmed the model and performed the experiments. SM collected and processed the epidemic data. HZ and LY were contributors in writing the manuscript. LM and TC revised the manuscript. All authors read and approved the final manuscript.

## Funding

This research was funded by National Key R&D Program of China (Grant No. 2021YFC2600500), National Natural Science Foundation of China (Grant No. 41771441), Key Basic Research Program of Shenzhen (Grant No. JCYJ20210324115411030), Major science and technology projects of Xinjiang Uygur Autonomous Region (Grant No. 2020A03004-4), and Natural Science Foundation of Guangdong Province (Grant No. 2021A1515011191).

## Conflict of Interest

The authors declare that the research was conducted in the absence of any commercial or financial relationships that could be construed as a potential conflict of interest. The handling editor WL declared a past co-authorship with the authors LY, TC, SF, HZ, and KL.

## Publisher's Note

All claims expressed in this article are solely those of the authors and do not necessarily represent those of their affiliated organizations, or those of the publisher, the editors and the reviewers. Any product that may be evaluated in this article, or claim that may be made by its manufacturer, is not guaranteed or endorsed by the publisher.
