# Supplementary material for: Combinational Recommendation of Vaccinations, Mask-Wearing, and Home-Quarantine to Control Influenza in Megacities: An Agent-Based Modeling Study With Large-Scale Trajectory Data
Source: Front Public Health. 2022 May 26;10:883624. doi: 10.3389/fpubh.2022.883624 (PMC9204335; doi:10.3389/fpubh.2022.883624)
Supplement: Supplementary file 1 [file Presentation_1.pdf]

Supplementary Material

1 Demographic and geographic data of Shenzhen

The *census data* of Shenzhen in 2010 includes the age distribution of the urban population, the sex ratio of each age group, occupations, family structure distribution, the number of families in each community, and the education levels of the individuals.

The *building census data* includes information on the location, height, area, and type (factories, educational buildings, residential buildings, office buildings, shopping malls, etc.) of each building in Shenzhen. In 2012, there were 608,305 buildings in Shenzhen City.

2 Agent-based mobility model

The whole urban population is synthesized according to the population size, gender, age structure, etc., from the census data. Then, according to the family structure and the number of families in each community in the census data, all synthetic individuals are assigned to different families, and the community code where the family address is located is given. The synthetic individuals are divided into individuals with and without mobile phones according to the mobile phone utilization rate at different ages.

The travel chains of individuals with mobile phones are constructed according to the mobile phone location data, and the travel chains of individuals without mobile phones are constructed according to the travel survey data. After cleaning the mobile phone location data, 5.8 million users with complete 24-hour records during the day were selected. For each mobile phone user, their home location and work location are identified based on the frequent places they stay during the day and at night. Then, taking the home address of mobile phone users as the matching object, a 24-hour activity chain is synthesized for each mobile phone user and given a workplace. The travel survey data includes individual travel records, home addresses, and workplaces. We match the home addresses of the synthesized individuals without mobile phones with the survey data and then synthesize their travel chains. Students' schools are allocated according to their family address.

3 Contact network

Taking the independent individuals in urban areas as agents, the agent-based mobility model we constructed has 11.16 million synthesized individuals (4.5 million households) and 230,000 work units in Shenzhen. Population age structure and household size in the model are shown in Fig. S2A and Fig. S2B, respectively. The activity chain of an agent is divided into 24 hours with an activity purpose and a series of active locations, and a typical activity chain is expressed in the form of "home-work-leisure-home". An individual has an activity location every hour, which is represented by building coordinates.

Based on the agent travel chain, with an hour as the time step, a contact network of 24 time series in a day is dynamically constructed. This study set individuals with the same activities at the same time and in the same building location as individuals with spatiotemporal co-occurrence. Individuals with spatiotemporal co-occurrence at home, work, and school are divided into multiple fixed contact groups. The individuals in the fixed contact group are regular encounters, and the other individuals outside the fixed contact group are random encounters, so as to simulate close contacts for acquaintances and casual contacts for strangers (Table S1). The comparison between the contact numbers of different ages in the model and the survey data of Shanghai (1) is shown in the Fig. S2C.

4 Effectiveness of three interventions

This study analyzed the effects of three interventions on adults (Fig. S3). Different vaccination rates for adults led to a gradual decline in the infection scale, with vaccination for 50% of adults reducing the percentage of infected individuals to 19.64% (Fig. S3A). During the influenza season, 70% of infected adults wear masks for all activities except living at home, reducing the percentage of infected people to 20.18% (Fig. S3B). The decreasing rate of infection size showed a linear trend with the increase in vaccine coverage rate (Fig. S3D), but the decreasing rate of infection size showed a non-linear trend with the increase in mask wearing rate (Fig. S3E). By improving the home-quarantine proportion of infected adults after onset(Fig. S3C), it is found that the decline rate of infection size shows a linear trend (Fig. S3F). The higher the home-quarantine proportion, the higher the decline rate of infection size (except when all infected adults are quarantined at home after onset).

5 Sensitivity to immune ratios

In order to understand the impact of the immunization ratio on the temporal and spatial diffusion characteristics of influenza, the model simulates two scenarios: a population immunization ratio of 0 (comparison scenario) and a population immunization ratio of 30% (baseline scenario). The two scenarios are simulated 100 times each, and the median value of the 100 simulation results is taken as the final simulation result. In this study, the effects of the initial immune ratio on the simulation results were analyzed from the aspects of effective reproduction number, cumulative infection size, peak time, infection location, age distribution of infected persons, and the infection size in each spatial unit at different spatial scales.

In the comparison scenario, 61.94% of the population in Shenzhen will be infected (Fig. S4A), and the peak time of the daily confirmed curve is 28 days ahead of the baseline scenario (Fig. S4B). The infection ratios of different age groups and the infection ratios of different activity types are basically consistent with the baseline scenario (Fig. S4C). At the scale of district, sub-district, and community, the infection sizes of the two scenarios have a strong linear relationship (Fig. S4D-F). The effective reproduction number in the baseline scenario is 1.59, while in the comparison scenario it is 2.39, which is greater than 2.0. It can be seen that different immune ratios have an impact on the infection size, peak time, and effective reproduction number, and they have a weak impact on the relative transmission intensity in each spatial unit, the infection ratio of different age groups, and the infection ratio of different activity locations in Shenzhen.

6 References

1. Zhang J, Klepac P, Read JM, Rosello A, Wang X, Lai S, et al. Patterns of human social contact and contact with animals in Shanghai, China. Scientific Reports. 2019;9(1):1-11.

7 Supplementary Figures

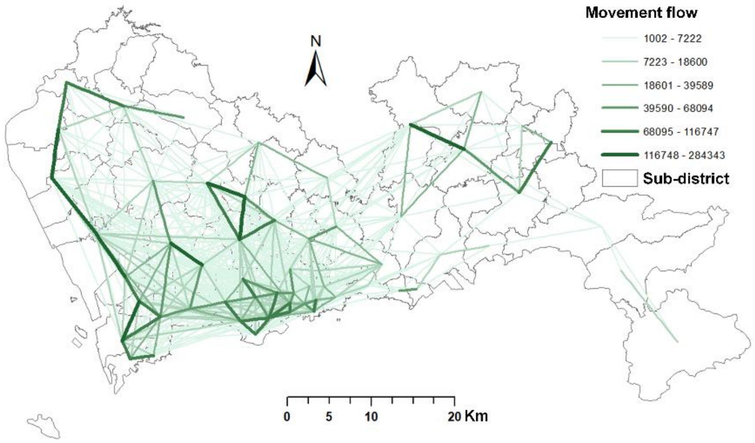

Supplementary Figure 1. The major movement flows of mobile phone users in Shenzhen.

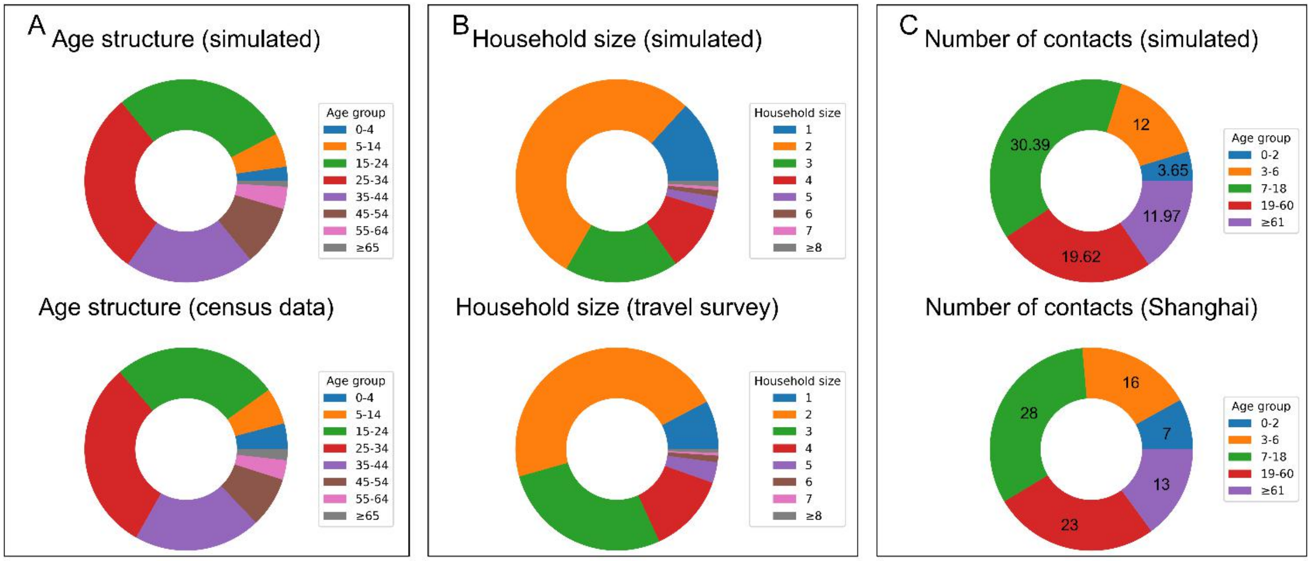

Supplementary Figure 2. Population attribute of Shenzhen. **(A)** Population age structure. **(B)** Household size. **(C)** Contact number of individuals of different ages.

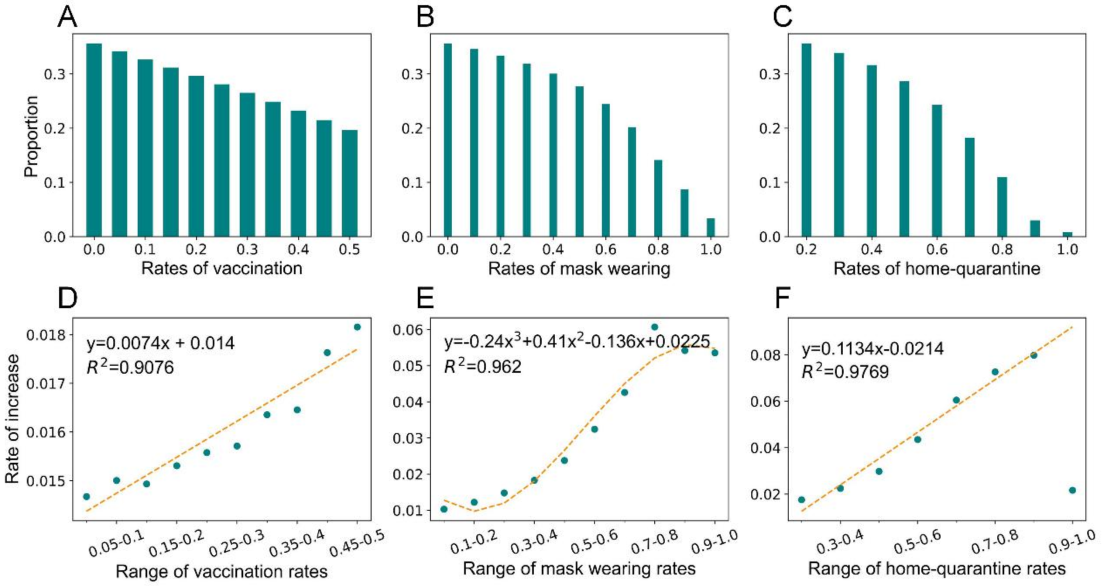

Supplementary Figure 3. Effectiveness of three interventions.

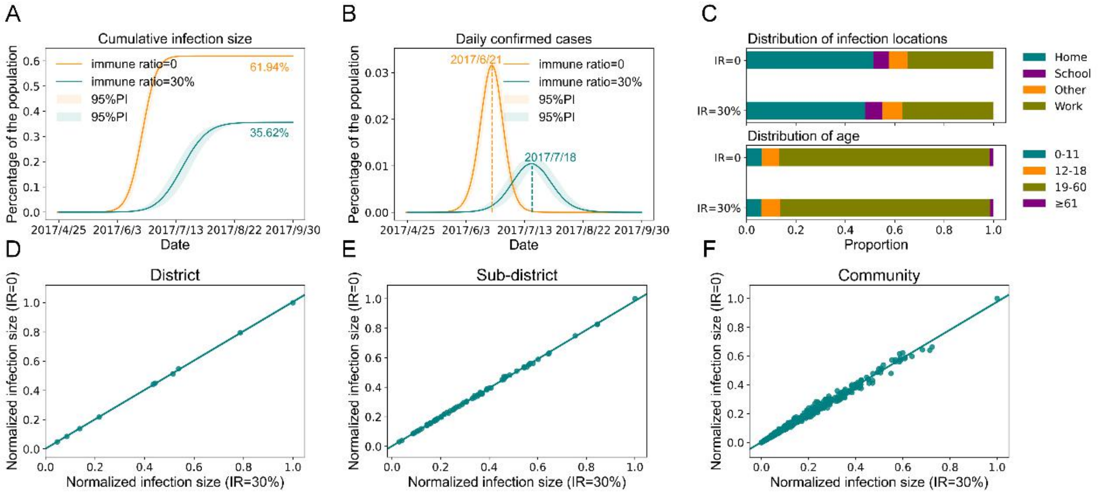

Supplementary Figure 4. Sensitivity analysis of immune ratio (IR stands for immune ratio). **(A)** Daily cumulative infection size. **(B)** Daily confirmed curve. **(C)** Infection locations and age distribution. **(D)**- **(F)** Correlation of infection size by spatial unit at the district, sub-district, and community levels.

8 Supplementary Tables

Supplementary Table 1. Contact types, daily contact number, and contact intensity of individuals

Activity type	Spatiotemporal co-occurrence	Number of fixed contacts	Number of close contacts	Number of casual contacts	Contact intensity
Home	Family members	Number of family members	Number of family members	0	0.37
School	Students in the same kindergarten	25 classmates	10 classmates	2 individuals in other classes	0.25
	Students in the same primary school	50 classmates	20 classmates	5 individuals in other classes	0.25
	Students in the same middle school	50 classmates	20 classmates	5 individuals in other classes	0.25
	Students in the same high school	50 classmates	20 classmates	5 individuals in other classes	0.25
Work	Colleagues in the same workplace	10 members in the working group	7 members in the working group	3 colleagues outside the working group	0.26
Other	Individuals engaged in the same activity in the same building	0	0	Restricted by the long tail distribution of the total contact number	0.1
